# Probiotics for Preventing Ventilator-Associated Pneumonia in Mechanically Ventilated Patients: A Meta-Analysis with Trial Sequential Analysis

**DOI:** 10.3389/fphar.2017.00717

**Published:** 2017-10-09

**Authors:** Hong Weng, Jian-Guo Li, Zhi Mao, Ying Feng, Chao-Yang Wang, Xue-Qun Ren, Xian-Tao Zeng

**Affiliations:** ^1^Center of Evidence Based and Translational Medicine, Zhongnan Hospital of Wuhan University, Wuhan, China; ^2^Department of Urology, Zhongnan Hospital of Wuhan University, Wuhan, China; ^3^Department of Critical Care Medicine, Zhongnan Hospital of Wuhan University, Wuhan, China; ^4^Department of Critical Care Medicine, Chinese PLA General Hospital, Beijing, China; ^5^Center of Evidence Based Medicine, Huaihe Hospital of Henan University, Kaifeng, China

**Keywords:** probiotics, ventilator-associated pneumonia, meta-analysis, trial sequential analysis, randomized-controlled trial

## Abstract

**Background and Objective:** Ventilator-associated pneumonia (VAP) is still an important cause of morbidity and mortality in mechanically ventilated patients. The efficacy of the probiotics for preventing VAP is still controversial. Present study was conducted to comprehensively evaluate the effect of probiotics on VAP prevention in mechanically ventilated patients.

**Methods:** PubMed, Embase, and CENTRAL were searched up to September 2016. Eligible trials designed with randomized controlled trials (RCTs) comparing probiotics with control in mechanically ventilated patients were included. Risk ratios (RRs) and mean differences (MDs) with 95% confidence intervals (CIs) were estimated with fixed or random effects models. Trial sequential analysis (TSA) was performed using TSA 0.9beta software.

**Results:** Thirteen RCTs (*N* = 1969) were included. Overall, probiotics were associated with reduced incidence of VAP (RR = 0.73, 95% CI = 0.60–0.89; *P* = 0.002), which was confirmed by TSA (TSA adjusted 95% CI = 0.55–0.96). However, no significant difference was observed in 90-day mortality (RR = 1.00, 95% CI = 0.72–1.37; *P* = 0.99), overall mortality (RR = 0.84, 95% CI = 0.70–1.02; *P* = 0.09), 28-day mortality (RR = 1.06, 95% CI = 0.72–1.57; *P* = 0.99), intensive care unit (ICU) mortality (RR = 0.97, 95% CI = 0.74–1.27; *P* = 0.82), hospital mortality (RR = 0.81, 95% CI = 0.65–1.02; *P* = 0.07), diarrhea (RR = 0.99, 95% CI = 0.83–1.19; *P* = 0.92), length of ICU stay (MD = −2.40 days, 95% CI = −6.75 to 1.95; *P* = 0.28), length of hospital stay (MD = −1.34 days, 95% CI = −6.21 to 3.54; *P* = 0.59), and duration of mechanical ventilation (MD = −3.32 days, 95% CI = −6.74 to 0.09; *P* = 0.06).

**Conclusions:** In this meta-analysis, we found that probiotics could reduce the incidence of VAP in mechanically ventilated patients. It seems likely that probiotics provide clinical benefits for mechanically ventilated patients.

## Introduction

Ventilator-associated pneumonia (VAP) is still an important cause of morbidity and mortality in mechanically ventilated patients even though the incidence thereof has been decreased in the past several years in America (Metersky et al., 2016). It is estimated that VAP may be responsible for ~27–47% of intensive care unit (ICU) acquired infections (Grap et al., 2012). The clinical and economic burden of VAP remains high and the application of existing VAP prevention strategies is variable but disappointing (Muscedere et al., 2008; Amin, 2009; Kallet, 2015). Therefore, a simple, inexpensive, and safe prevention strategy will contribute to the decrease of VAP occurrence rate and corresponding burden. The pathogenesis of VAP is complicated; however it typically involves the colonization of upper aerodigestive tract with pathogenic bacteria and the leakage of contaminated oropharyngeal secretions into the lung (Kollef, 2005; Baselski and Klutts, 2013). Numerous studies have assessed various strategies of VAP prevention which can be classified into pharmacologic and non-pharmacologic interventions. Compared to other strategies, probiotics have been considered as a new intervention for VAP prevention in critical care medicine.

In recent years, several studies suggest that orally administered probiotics may conduce to the prevention of VAP (Siempos et al., 2010; Theodorakopoulou et al., 2013). However, the conclusions on this topic are still controversial (Siempos et al., 2010; Gu et al., 2012; Wang et al., 2013; Bo et al., 2014). In 2010, Siempos et al. (2010) performed a meta-analysis with five trials and supported that probiotics were associated with decreased risk of VAP, which was further confirmed by a Cochrane systematic review with eight trials (Bo et al., 2014). However, another meta-analysis carried out by Gu et al. (2012) with seven trials concluded that probiotics were not beneficial to mechanically ventilated patients. Additionally, the results of a subsequent meta-analysis performed by Wang et al. (2013) with five trials also demonstrated that probiotics had no beneficial effect for prevention of VAP. Several trials have been applied to assess the role of probiotics in VAP prevention since the previous meta-analyses were published. Additionally, due to uncertain efficacy and safety of probiotics, most ICU pharmacists would not currently recommend this strategy for prevention of VAP (Wheeler et al., 2016). Therefore, we performed an updated meta-analysis to evaluate the effectiveness and safety of probiotics for preventing VAP, thereby providing a more precise evidence for clinical practice.

## Methods

### Eligibility criteria

This meta-analysis is reported based on the methodology of Cochrane Handbook (Higgins and Green, 2011) and conducted in adherence to Preferred Reporting Items for Systematic Reviews and Meta-Analyses (PRISMA) statement (Moher et al., 2009). The inclusion criteria were a s following: (1) patients: the study subjects were mechanically ventilated patients; (2) intervention: probiotics; (3) comparison: placebo or other drugs; (4) outcomes: primary outcome was incidence of VAP; secondary outcomes were 90-day mortality, overall mortality, 28-day mortality, ICU mortality, and hospital mortality; tertiary outcomes were diarrhea, length of ICU stay, length of hospital stay, and duration of mechanical ventilation; (5) study type: only randomized controlled trials (RCTs) that were peer-reviewed and available in full-text would be included in this meta-analysis.

### Search strategy

PubMed, Embase, and CENTRAL on the Cochrane Library were comprehensively searched for all relevant RCTs up to September 2016 by two authors (HW and JL). The following items were combined and adopted to retrieve original studies: “probiotic,” “probiotics,” “prebiotic,” “prebiotics,” “symbiotic,” “synbiotics,” “lactobacillus,” “lactobacilli,” “bifidobacterium,” “pneumonia,” “random,” “placebo,” and “trial.” Reference lists of relevant reviews or meta-analyses were manually searched. No language restriction was applied. Any discrepancy was solved by consensus or discussion with a third author (XZ) when necessary.

### Data extraction and risk of bias assessment

Two reviewers (HW and JGL) independently extracted data from eligible studies using a pre-specified data extraction form and assessed the risk of bias of included studies. The extracted information: included name of first author, year of publication, country, institutions, language, funding source, characteristic of participants, details of intervention and comparison treatment, definition of VAP, outcomes, and methodological design. Discrepancy was solved by negotiation between them. The risk of bias of included studies was assessed according to Cochrane Handbook for Systematic Reviews of Interventions criteria (Higgins and Green, 2011).

### Statistical analysis

Dichotomous outcome variables were measured using risk ratios (RRs) and corresponding 95% confidence intervals (CIs). Continuous outcome variables were measured using mean differences (MDs) and corresponding 95% CIs. Heterogeneity between studies was detected by Cochrane's *Q*-test with *P* < 0.1 as a significance level, and quantitatively measured through *I*^2^ statistic. Fixed effects model was applied to perform the meta-analysis if the *P*-value of Cochrane's *Q*-tests was more than 0.1, otherwise, random effects model was utilized. The statistical significance level was set at 0.05 for this meta-analysis. All the data syntheses were accomplished using RevMan 5.3 software. The number needed to treat (NNT) was also estimated for primary outcome. Sensitivity analyses were performed by excluding studies which would confound the results.

Cumulative meta-analyses of RCTs are at risk of yielding random errors due to sparse data and repetitive testing of accumulating data (Wetterslev et al., 2017). Trial sequential analysis (TSA) depends on the quantification of the required information size (RIS), i.e., optimal information size. TSA was undertaken using TSA 0.9 beta software if the number of included trials was more than five. The RIS was estimated using relative risk reduction and heterogeneity adjusted information size for dichotomous outcomes (Brok et al., 2008; Wetterslev et al., 2008; Thorlund et al., 2009). The result was confirmed as true positive if the cumulative Z-curve surpassed the Lan-DeMets trial sequential monitoring boundary or reached the RIS above the conventional significance level line (*Z* = 1.96); and the result was confirmed as true negative if the cumulative Z-curve reached the futility boundary or reached the RIS below the conventional significance level line (*Z* = 1.96). TSA adjusted 95% CIs were also presented.

## Results

### Characteristics and risk of bias assessment of included trials

**Figure 1:**
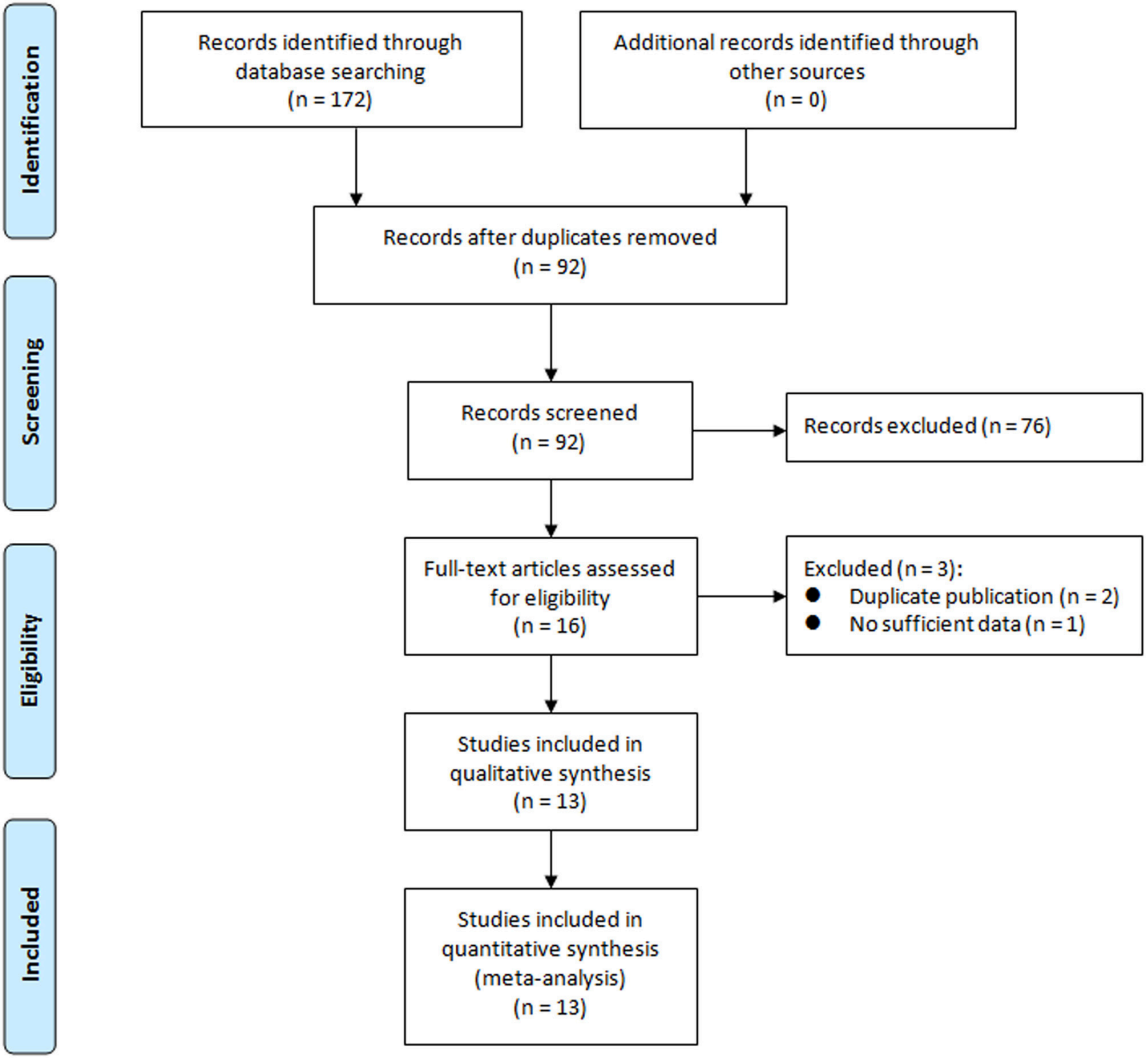
Flowchart of study selection process.

**Figure 2:**
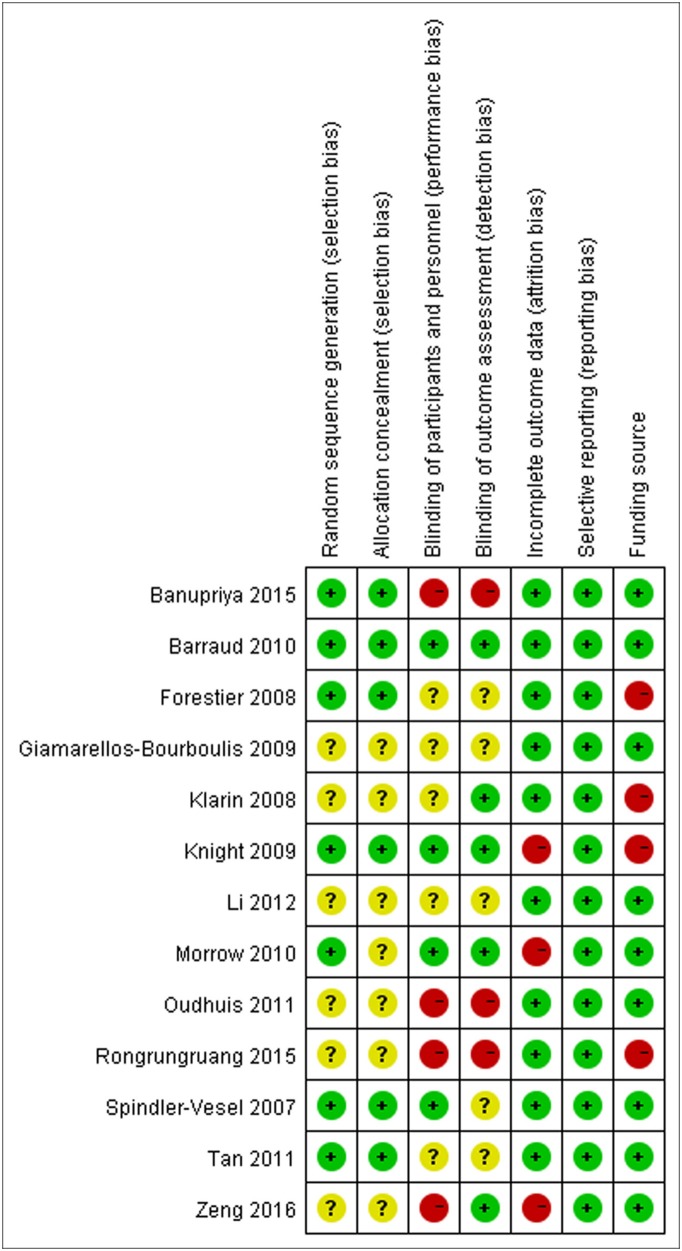
Risk of bias assessment of included trials.

We initially retrieved a total of 172 studies from the above-mentioned databases. After strict screening according to inclusion criteria, 13 RCTs (Spindler-Vesel et al., 2007; Forestier et al., 2008; Klarin et al., 2008; Giamarellos-Bourboulis et al., 2009; Knight et al., 2009; Barraud et al., 2010; Morrow et al., 2010; Oudhuis et al., 2011; Tan et al., 2011; Li et al., 2012; Banupriya et al., 2015; Rongrungruang et al., 2015; Zeng et al., 2016) were included in the present meta-analysis. The study selection process is presented in Figure 1. Characteristics of included trials are shown in Table 1. These trials were published between 2007 and 2016. The sample sizes of included trials were ranged from 35 to 259 (total number was 1,969). Two studies (Li et al., 2012; Banupriya et al., 2015) focused on children and one study (Klarin et al., 2008) only included probiotics as oral care. These three studies might confound the results of the overall analysis and sensitivity analyses were undertaken by removing these trials for relevant outcomes. Risk of bias assessment of included trials is displayed in Figure 2.

**Table 1:** Characteristics of included trials.

**Study**	**Setting**	**Participant**	**Intervention**		**Definition of VAP**
			**Probiotic group**	**Control group**	
Spindler-Vesel et al., 2007	A 20-bed university surgical ICU, Ljubljana, Slovenia	Multiple injured patients with an ISS of 18 and at least a 4 days ICU stay; *n* = 113	Nutricomp standard (B. Braun) 3.7 g protein, 13.7 g carbohydrate, 3.3 g fat per 100 mL. Patients in this group also received a supplement of a synbiotic consisting of 10^10^ Pediococcus pentosaceus 5–33:3, 10^10^ Lactococcus raffinolactis 32–77:1, 10^10^ *Lactobacillus paracasei* subsp paracasei 19, 10^10^ *Lactobacillus plantarum* 2,362 and 2.5 g of each of the following 4 fibers: β glucan, inulin, pectin, and resistant starch per sachet (Synbiotic 2000; Medipharm Kagerod, Sweden andDes Moines, IA). The contents of the sachets were dissolved in 100 mL of lukewarm sterile water, mixed carefully, and then added separately, before feeding was started	3 arms: Alitraq (Abbott-Ross, Abbott Park, IL) 5.25 g protein, 16.5 g carbohydrate, 1.55 g fat and 1.55 g glutamine, 446 mg arginine, 154 mg α-linolenic acid per 100 mL; Nova Source (Novartis Medical Nutrition, Basel, Switzerland) 4.1 g protein, 14.4 g carbohydrate, 3.5 g fat, 2.2 g fermentable fibers as fermentable guar gum per 100 mL; Nutricomp peptide (B. Braun, Melsungen, Germany) 4.5 g hydrolyzed protein, 16.8 g carbohydrate, 1.7 g fat per 100 mL	Microbiological specimens were collected and nosocomial infections were recorded as recommended by the Centers for Disease Control and Prevention and consensus conferences on ventilator-associated pneumonia
Forestier et al., 2008	A 17-bed ICU in the teaching hospital of Clermont-Ferand, France; 1 center	Patients aged 18 years or older with a stay longer than 48 h and a nasogastric feeding tube; *n* = 208	L. casei rhamnosus (10^9^ CFU) twice daily through a double-lumen nasogastric suction tube or orally, after removal of the tube, from the third day after admission to the ICU until discharge or death	Placebo (growth medium without bacteria); the method of administration was the same as the treatment group	The criteria require there to be at least 1 positive sample (protected specimen brush or plugged telescoping catheter for broncho-alveolar minilavage (>10^3^ CFUs/ml)) or endotracheal aspirate with (>10^5^ CFUs/ml and >25 leucocytes/high-power field); also required is the presence of 1 or several new abnormal radio graphical and progressive parenchymatous infiltrates and 1 of the following signs: purulent sputum production, fever (temperature > 38.5°C), pathogenic bacteria in blood culture without other infection source and bronchoalveolar minilavage with more than 5% cells with intracellular bacteria
Klarin et al., 2008	1 ICU; 1 center, Department of Anesthesiology and Intensive Care, University Hospital, Lund, Sweden	Patients with 18 years of age or older and critically ill with an anticipated need for mechanical ventilation of at least 24 h; *n* = 44	Initial mechanical steps were the same as in the control group but subsequent cleansing was instead performed with gauze swabs soaked in carbonated bottled water, after which Lp299 was applied to the mucosal surface of the oral cavity. 10 ml of a solution containing a total 10^10^ CFUs of Lp299 were used	Treated according to the department's standard protocol. Dental prostheses were removed; secretions were removed by suction; teeth were brushed using toothpaste; all mucosal surface were cleansed with swabs that had been moistened with a 1 mg/ml chlorhexidine solution	A new, persistent or progressive infiltrate on chest radiograph combined with at least 3 or the other 4 criteria; a purulent tracheal aspirate; positive culture of tracheal aspirates occurring after 48 h of mechanical ventilation; rectal or urine bladder temperature higher than 38.0°C or <35.5°C; WBC count more than 12 or <3
Giamarellos-Bourboulis et al., 2009	5 surgical ICUs of the Thessalomiki University's tertiary-care AHEPAHospitals and the affiliated 424th Military Hospital, Greece	Trauma patients; severe multiple organ injuries necessitating emergency tracheal intubation and ventilation support and subsequent hospitalization in ICU; *n* = 72	The synbiotic preparation (Synbiotic 2000 Forte, Medipharm, Sweden) consisted of a combination of 10^11^ CFU of each of four probiotics; Pediococcus pentoseceus 5–33:3, Leuconostoc mesenteroides 32–77:1, *L. paracasei* ssp 19, and *L. plantarum* 2362, as well as 2.5 g each of inulin, oat bran, pectin, and resistant starch. It was given in doses of 12 g (1 sachet) per day for a 15-day study period, diluted in 100 ml of tap water	The placebo preparation consisted of identical doses of powdered glucose polymer (maltodextrin, Caloreen, Nestle, UK)	New or persistent consolidation in lung X-ray; purulent tracheobronchial secretion; and clinical pulmonary infection score of more than 6
Knight et al., 2009	A 14-bedded general ICU of a 1400-bedded UK tertiary care University Hospital; 1 center	All intubated adult patients under mechanical ventilation for a minimum of 48 h and with no contraindications to enteral nutrition; *n* = 259	at least 2 days (4 doses in 48 h) of Synbiotic 2000 FORTE® (Medipharm, Kagerod, Sweden and Des Moines, IA), twice a day. Synbiotic 2000 FORTE® contains Pediococcus pentosaceus, Leuconostoc mesenteroides, *Lactobacillus paracasei* subsp paracasei and *Lactobacillus plantarum* (at a dose of 10^10^ bacteria per sachet) as probiotics and Betaglucan, Inulin, Pectin and Resistant starch (2.5 g of each) as prebiotics. Synbiotic was dissolved in 50–100 ml of sterile water and given as a bolus through a nasogastric/orogastric tube	Crystalline cellulose-based placebo. Placebo was dissolved in 50–100 ml of sterile water and given as a bolus through a nasogastric/orogastric tube	VAP was suspected if there was new progressive, or persistent (24 h), infiltration on chest radiograph plus at least 2 of the following: (1) temperature 38.0°C, (2) leucocytosis (WBC count >12 × 10^3^ μL^−1^) or leukopenia (WBC count <4 × 10^3^ μL^−1^), (3) purulent tracheobronchial secretions. All suspected cases were reviewed with appropriate clinical, radiological and sequential microbiological data (tracheal aspirates and bronchoalveolar lavage). Diagnosis was made prospectively and only confirmed if a blinded microbiologist and intensive care physician agreed on the diagnosis. Pneumonia was classified as VAP when diagnosed 48 h after intubation
Barraud et al., 2010	A medical intensive care unit, France; 1 center	All intubated adult patients under mechanical ventilation for a predicted period of at least 2 days; *n* = 149	Treatment consisted of the administration of 5 Ergyphilus® (Nutergia, Capdenac, France) capsules once a day. Ergyphilus® capsules consisted of a multi-species probiotic preparation containing 2 × 10^10^ of revivable bacteria (mainly *Lactobacillus rhamnosus* GG but also *Lactobacillus casei, Lactobacillus acidophilus* and *Bifidobacterium bifidum*). Treatment was diluted in 20 ml of water and administered daily by the nurse through the enteral feeding tube for the entire period of mechanical ventilation (but for a duration not exceeding 28 days). After weaning from the ventilator, treatment was given for 2 additional days and then stopped in the case of successful extubation, or continued in the case of extubation failure	Placebo capsules only contained the excipient	VAP was defined by the presence of: (1) a new and persistent infiltrate on chest radiograph associated with at least one of the following: purulent tracheal secretions, temperature 38.3°C or higher, and a leukocyte count of 10,000 μL^−1^ or higher; and (2) positive quantitative cultures of distal pulmonary secretions obtained from bronchoalveolar lavage (significant threshold more than 104 colonyforming units/mL)
Morrow et al., 2010	A 325-bed, university-based hospital that provides level 1 trauma services, USA	Adults at least 19 years old (the age of majority in Nebraska) were eligible for enrolment if the lead investigator and the treating physician agreed that there was a 95% likelihood that the patient would require mechanical ventilation with an endotracheal tube for at least 72 h; *n* = 138	Patients randomized to probiotic therapy received 2 × 10^9^ CFU of *Lactobacillus rhamnosu*s GG on a twice-daily basis. The contents of one capsule containing 10^9^ CFU of *Lactobacillus* were suspended in sterile, water-based surgical lubricant and administered as a slurry to the oropharynx; the contents of a second capsule containing 109 CFU of *Lactobacillus* were suspended in sterile water and given through the nasogastric tube	The same methods were used to deliver the contents of identical appearing capsules containing the inert plant starch inulin to patients randomized to placebo	According to the ACCP clinical criteria, quantitative cultures of distal airways samples were obtained by non-bronchoscopic bronchoalveolar lavage using a protected catheter (Combicath; KOL Biomedical Instruments, Chantilly, VA). The ACCP clinical criteria require a new and persistent infiltrate on chest radiographs with 2 of 3 supporting findings: fever (> 38.5 °C or, < 35.0°C), leukocytosis (white blood cells < 10,000/mm^3^ or < 3000/mm^3^) and purulent sputum
Oudhuis et al., 2011	Consecutive patients admitted to the ICU at the Maastricht University Medical Centre (705 beds) and the Atrium Medical Centre Heerlen (a 545-bed teaching hospital)	Patients were older than 18 years, and had expected duration of mechanical ventilation of at least 48 h, expected length of ICU stay of at least 72 h, or both; *n* = 254	Patients received a solution of viable *Lactobacillus plantarum* 299/299v in a dose of 5 × 10^9^ CFU together with 6 g of rose-hip (Probi AB, Lund, Sweden). The manufactured freeze-dried powder was dissolved in 75 ml of water and applied two times daily through a nasogastric tube. Administration of study product was continued by nasogastric tube until ICU discharge, death or final removal of the tube	Selective decontamination of the digestive tract. Four times daily an oral paste (polymyxin E, gentamicin, amphotericin B), enteral solution (same antibiotics), intravenous injection cefotaxime (first 4 days)	Confirmation of clinically suspected VAP required ≥ 2% cells containing intracellular organisms and/or a quantitative culture result of ≥ 10^4^ CFU/ml in bronchoalveolar lavage fluid
Tan et al., 2011	6-bed specialized ICU, Department of Neurosurgery, Affiliated Hospital of North Sichuan Medical College, Nanchong, China	Closed head injury alone; admission within 24 h after trauma; a Glasgow Coma Scale score between 5 and 8; aged 18–60 years old; and able to be fed via nasogastric tube within 48 h after admission; *n* = 35	Participants received enteral nutrition within 48 h following hospital admission by nasogastric tube. Golden Bifid (Shuangqi Pharmaceutical Co., Ltd, InnerMongolia, China) 0.5 × 10^8^ *Bifidobacterium longum*, 0.5 × 10^7^ *Lactobacillus bulgaricus* and 0.5 × 10^7^ *Streptococcus thermophilus*, dissolved in 20 ml sterilized, distilled water and administered through a nasogastric tube for 21 consecutive days, 7 sachets administered BID at 7am, 3pm and 11pm (total 10^9^)	Participants received enteral nutrition within 48 h following hospital admission by nasogastric tube. Continued to receive enteral nutrition (3.8 g protein, 13.8 g carbohydrate, 3. 4 g fat/100 ml, osmolarity 250 mOsm/l, no fibers; Ruisu, Huarui Pharmaceutical Co., Ltd, Beijing, China)	VAP was defined as pneumonia occurring more than 48 h after endotracheal intubation, and was diagnosed by the presence of both a new or progressive radiographic infiltrate plus at least two clinical features—fever > 38.0°C, leucocytosis (white blood cells count > 12 × 10^9^/l), leucopenia (white blood cells count < 4 × 10^9^/l), or purulent tracheobronchial secretions—and positive semiquantitative cultures of tracheobronchial secretions
Li et al., 2012	A medical intensive care unit, China; 1 center	Neonates with an anticipated need for mechanical ventilation of at least 48 h; *n* = 165	The probiotics group was administered with oral probiotics in addition to routine treatment. Live combined bifidobacterium, lactobacillus and enterococcus $powderle viable (Xinyi Pharmaceutical Co., Ltd, Shanghai, China) 0.5 × 10^8^ CFU *Bifidobacterium longum*, 0.5 × 10^7^ CFU *Lactobacillus bulgaricus* and 0.5 × 10^7^ *Enterococcus faecalis*	Routine treatment	VAP was defined by the presence of: (1) purulent tracheobronchial secretion more than 48 h after endotracheal intubation; (2) a new or progressive infiltrate on chest radiograph; (3) fever and leucocytosis (WBC count > 10 × 10^3^ μL^−1^)
Banupriya et al., 2015	A 12-bed PICU of a tertiary care teaching hospital, India	All children aged 12 years or less admitted to PICU and who were likely to need mechanical ventilation for more than 48 h were recruited; *n* = 150	Probiotic capsules containing 2 billion CFU of *Lactobacillus*, 1 billion CFU of *Bifidobacterium*, and 300 million CFU of *Streptococcus thermophilus* were used. One probiotic capsule contained a total of 3.3 billion CFU of probiotic organisms. One capsule was administered twice a day mixed with milk (or 5 ml of 5% dextrose solution if enteral feeding had not been started) and given through a nasogastric tube. A total of 6.6 billion CFU of probiotic organisms per day was administered to each child in the probiotic group for the initial 7 days or till discharge, whichever was earlier	Standard care, no placebo	VAP was defined as a new (developing more than 48 h after the start of mechanical ventilation or within 48 h of extubation) or persisting radiographic infiltrate (persisting radiographically for at least 72 h) that develops in conjunction with one of the following: (1) Radiographic evidence of pulmonary abscess formation (i.e., cavitations within pre-existing pulmonary infiltrates); (2) Two of the following: fever (increase in the core temperature of at least 1°C and a core temperature of above 38.3°C), leukocytosis (25% increase in circulating leukocytes from baseline/a leukocyte count of >10,000/mm^3^), and purulent tracheal aspirate [Gram's stain showed more than 25 neutrophils per high-power field (× 400 magnification)]; (3) A positive blood or pleural fluid culture with the microorganisms recovered from blood or pleural fluid cultures being identical to the organisms recovered from cultures of respiratory secretions
Rongrungruang et al., 2015	A 2300-bed teritary care university hospital in Bangkok	The study subjects were adult hospitalized medical patients who were expected to receive mechanical ventilation at least 72 h and had no VAP at enrollment; *n* = 147	The patients in the probiotics group received 80 ml of commercially-available fermented dairy product containing 8 × 10^9^ colony-forming units (cfu) of *Lactobacillus casei* (Shirota strain) (Yakult®) for oral care after the standard oral care once daily. An additional 80 ml of the aforementioned fermented dairy product was given via enteral feeding once daily for 28 days or when their endotracheal tubes were removed. Probiotics was discontinued when diarrhea related to probiotics occurred	The patients in the control group did not receive any additional products	A diagnosis of VAP was made if the patient had a new, persistent, or progressive infiltrate visible on a chest radiograph in combination with at least 3 of the following 4 criteria: (1) body temperature greater than 38°C or < 35.5°C, (2) leukocytosis (>10,000 leukocytes/mm^3^) or leukopenia (<3,000 leukocytes/mm^3^), (3) purulent tracheal aspirate, and (4) a semi-quantitative culture of tracheal aspirate samples that was positive for pathogenic bacteria
Zeng et al., 2016	11 participating ICUs in nine Chinese teaching hospitals	All critically ill adult patients (age ≥ 18 years) with an expected need of mechanical ventilation for at least 48 h were eligible for entry into the study; *n* = 235	The probiotic group was given commercially available probiotics capsules (Medilac-S, China) 0.5 g three times daily plus standard preventive strategies of VAP. Patients in the probiotics group started taking the capsules within 2 h after randomization. Each probiotics capsule contained active *Bacillus subtilis* and *Enterococcus faecalis* at a concentration of 4.5 × 10^9^/0.25 g and 0.5 × 10^9^/0.25 g, respectively. All patients had a nasogastric tube. For delivery to the patient, the probiotics capsules were first broken open and the contents diluted in 50–80 ml sterile water; this solution was administered as a bolus through a nasogastric tube by the nursing staff. All probiotics capsules were stored at 4°C	The control group received standard preventive strategies only. The standard preventive strategies of VAP included daily screening for weaning potential and weaning from mechanical ventilation as soon as possible, hand hygiene, aspiration precautions, and prevention of contamination	A clinical diagnosis of VAP was based on the presence of a new, persistent or progressive infiltrate on chest radiographs that persisted for at least 48 h (as interpreted by radiologists blinded to the patients' treatment assignments) combined with at least two of the following criteria: (1) a temperature of > 38.0°C or < 35.5°C; (2) a blood leukocytosis count of > 12 × 10^3^/mm^3^ or < 3 × 10^3^/mm^3^ and/or left shift; (3) purulent tracheal aspirates. All clinical diagnoses of VAP were evaluated and agreed upon by two of the authors

*ISS, Injury Severity Score; ICU, intensive care unit; CFU, colony-forming units; ACCPA, American College of Chest Physicians; PICU, pediatric intensive care unit; VAP, ventilator associated pneumonia*.

### Primary outcome: incidence of VAP

**Figure 3:**
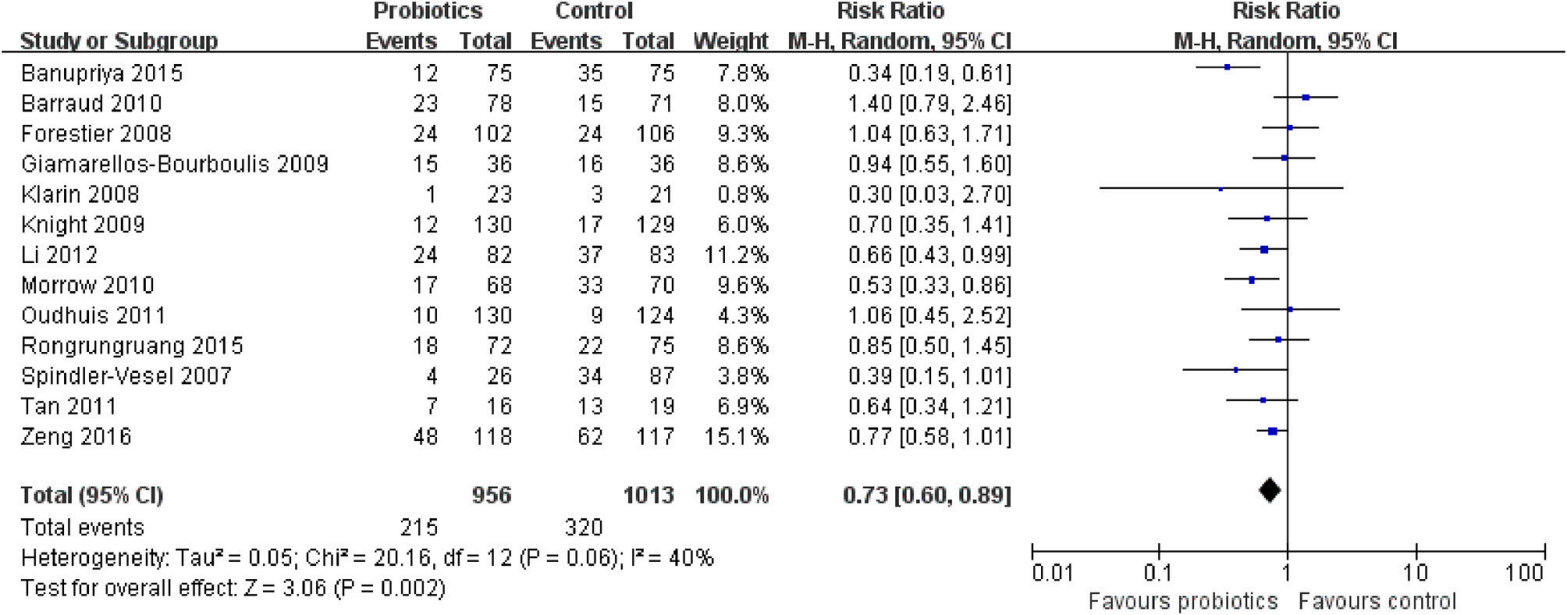
Forest plot of incidence of VAP.

**Figure 4:**
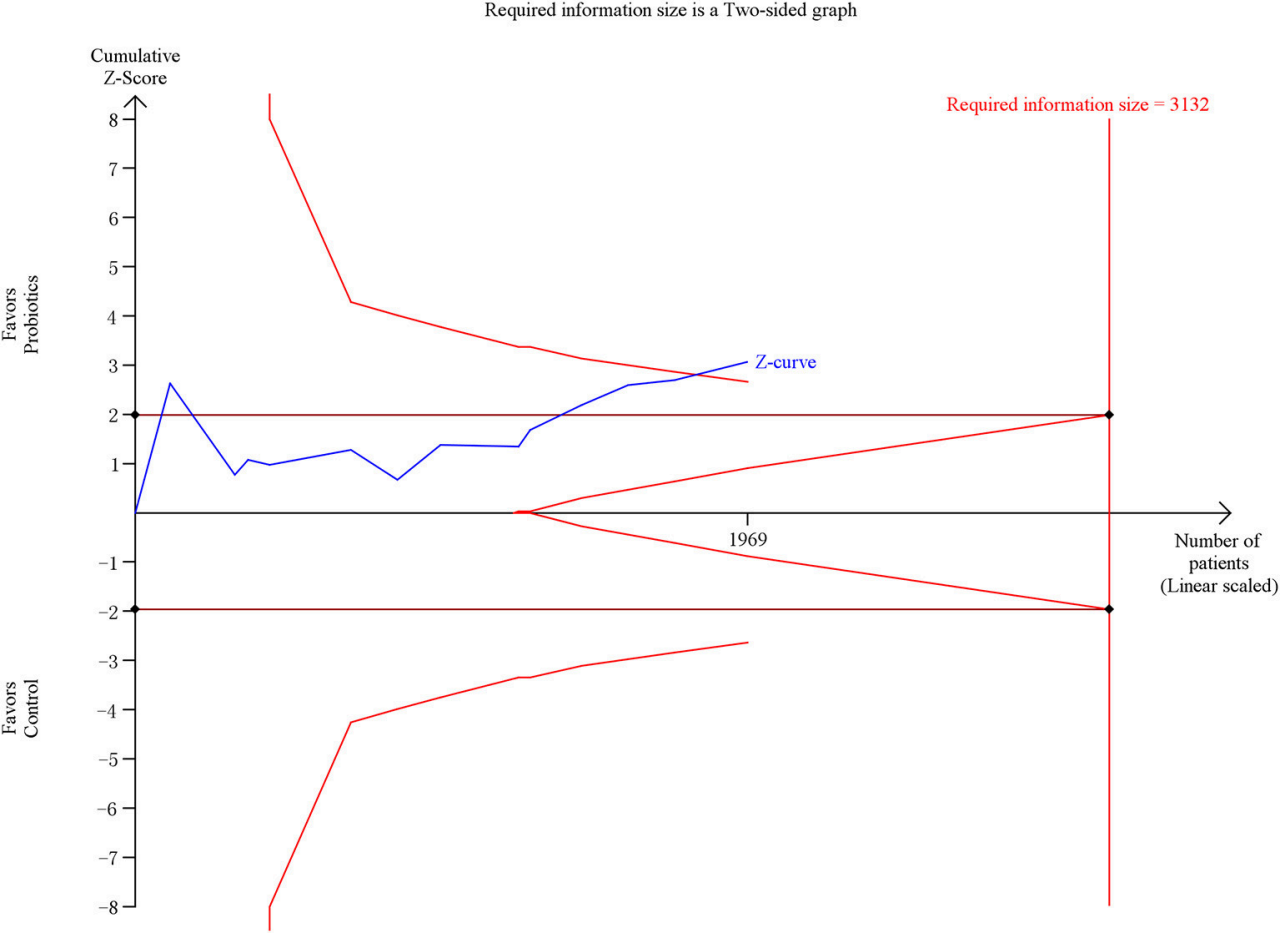
Trial sequential analysis of VAP.

The meta-analysis involving 13 trials (1,969 patients) showed a significantly decreased risk in incidence of VAP in patients exposed to probiotics based on random-effects model (RR = 0.73, 95% CI = 0.60–0.89; *P* = 0.002), as demonstrated in Figure 3. Low to moderate between-study heterogeneity was detected (*P* = 0.06, *I*^2^ = 40%). The NNT was 10.9 (95% CI = 7.7–19.3). The TSA adjusted 95% CI ranged from 0.55 to 0.96. The TSA result showed that 1,969 (62.9%) of the RIS of 3,132 patients was accrued. The cumulative z-curve crossed the conventional boundary for benefit and crossed the trial sequential monitoring boundary for benefit (Figure 4), indicating that firm evidence of probiotics for preventing VAP was obtained. Sensitivity analysis by removing three trials (Klarin et al., 2008; Li et al., 2012; Banupriya et al., 2015) showed similar results to the overall analysis (RR = 0.86, 95% CI = 0.66–0.97; *P* = 0.02).

### Secondary outcome 1a: 90-day mortality

**Figure 5:**
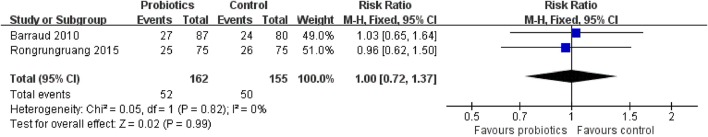
Forest plot of incidence of 90-day mortality.

Two trials concerning 317 patients presented follow-up data up to 90 days. The meta-analysis of these two trials showed no significant difference in 90-day mortality in patients exposed to probiotics based on fixed-effects model (RR = 1.00, 95% CI = 0.72–1.37; *P* = 0.99), as revealed in Figure 5. No evidence of between-study heterogeneity was detected (*P* = 0.94, *I*^2^ = 0%).

### Secondary outcome 1b: overall mortality

**Figure 6:**
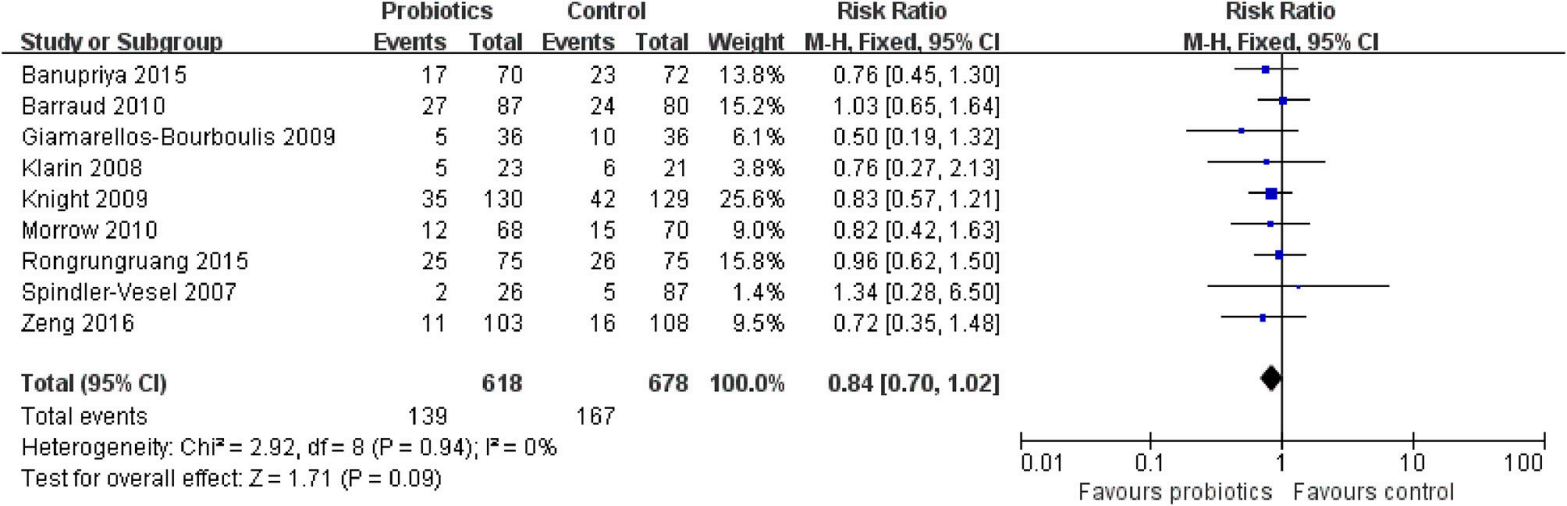
Forest plot of incidence of overall mortality.

**Figure 7:**
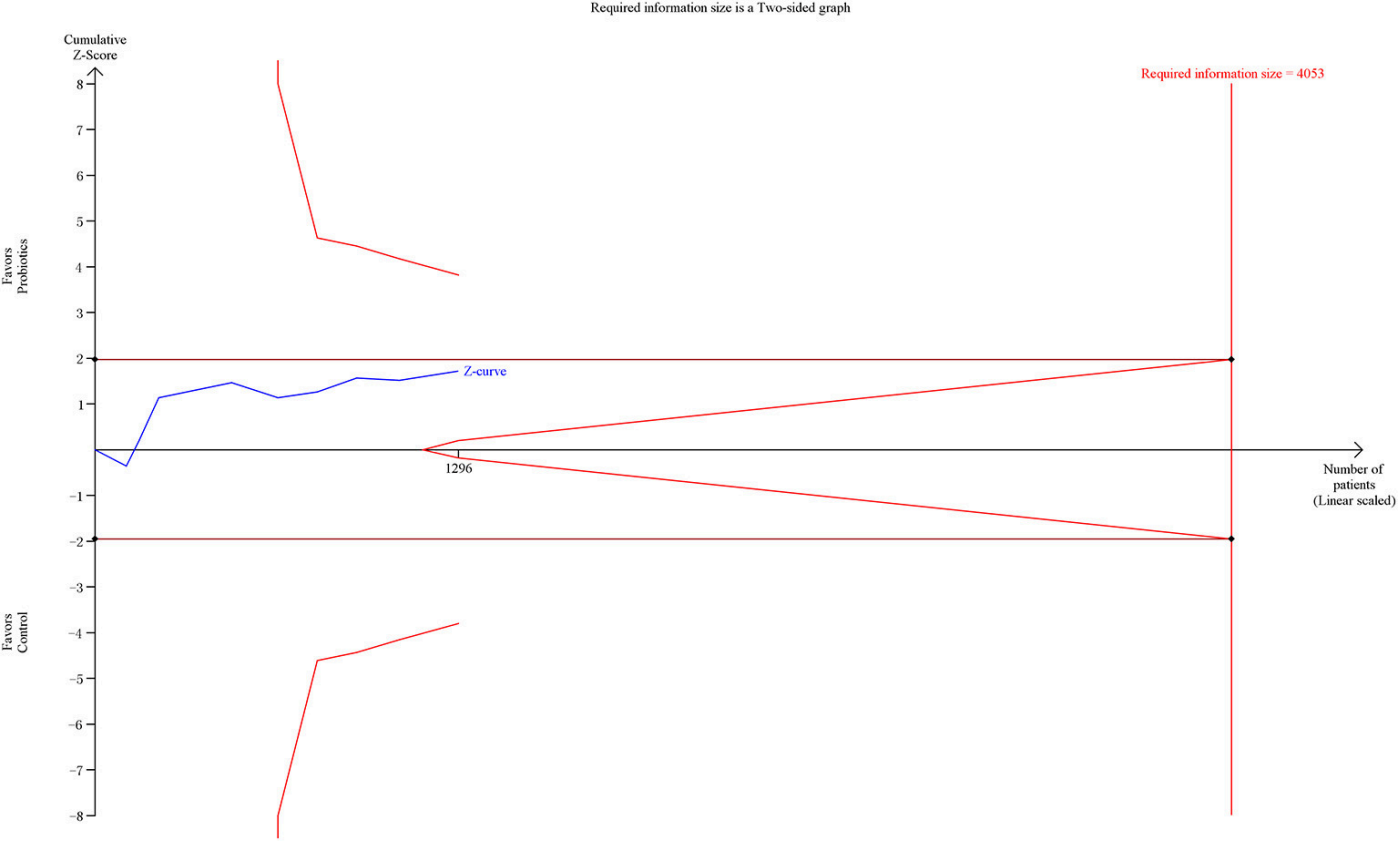
Trial sequential analysis of overall mortality.

Overall mortality data were obtained from nine RCTs involving 1,296 patients. The meta-analysis of these nine trials indicated no significant difference in overall mortality in patients exposed to probiotics based on fixed-effects model (RR = 0.84, 95% CI = 0.70–1.02; *P* = 0.09), as shown in Figure 6. No evidence of between-study heterogeneity was detected (*P* = 0.94, *I*^2^ = 0%). The TSA adjusted 95% CI was ranged from 0.58 to 1.23. The TSA result showed that 1,296 (32.0%) of the RIS of 4,053 patients was accrued. The cumulative z-curve crossed neither the conventional boundary for benefit nor the trial sequential futility boundary for benefit (Figure 7), suggesting that the current evidence was inconclusive. Sensitivity analysis by removing two trials (Klarin et al., 2008; Banupriya et al., 2015) showed similar results to the overall analysis (RR = 0.86, 95% CI = 0.70–1.07; *P* = 0.17).

### Secondary outcome 1c: 28-day mortality

**Figure 8:**
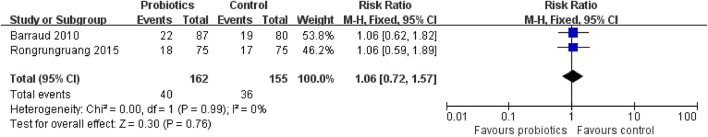
Forest plot of incidence of 28-day mortality.

Two trials with 317 patients presented follow-up data up to 28 days. The meta-analysis of these two trials showed no significant difference in 28-day mortality in patients exposed to probiotics based on fixed-effects model (RR = 1.06, 95% CI = 0.72–1.57; *P* = 0.99), as displayed in Figure 8. No evidence of between-study heterogeneity was detected (*P* = 0.99, *I*^2^ = 0%).

### Secondary outcome 1d: ICU mortality

**Figure 9:**
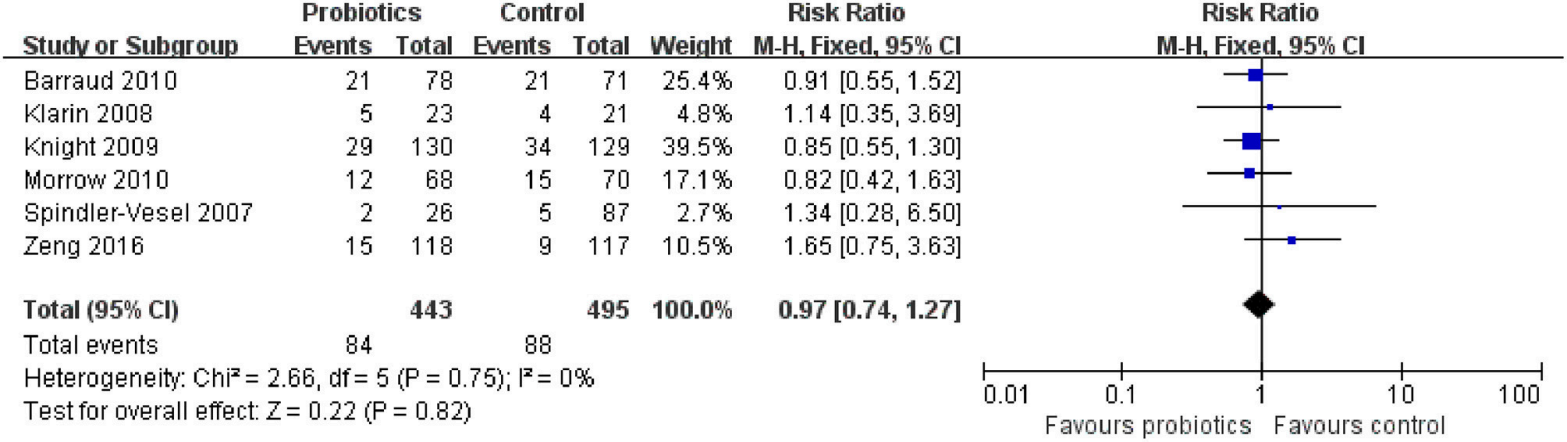
Forest plot of incidence of ICU mortality.

**Figure 10:**
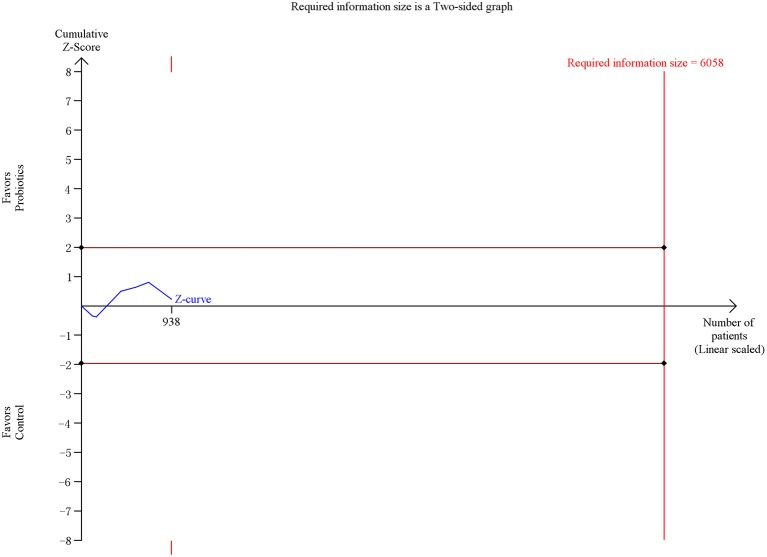
Trial sequential analysis of ICU mortality.

Six trials including 938 patients reported the ICU mortality data. The meta-analysis of these six trials exhibited no significant difference in ICU mortality in patients exposed to probiotics based on fixed-effects model (RR = 0.97, 95% CI = 0.74–1.27; *P* = 0.82), as shown in Figure 9. No evidence of between-study heterogeneity was detected (*P* = 0.75, *I*^2^ = 0%). The TSA adjusted 95% CI was ranged from 0.33 to 2.87. The TSA result showed that 938 (15.5%) of the RIS of 6,058 patients was accrued. The cumulative z-curve crossed neither the conventional boundary for benefit nor the trial sequential futility boundary for benefit (Figure 10), revealing that the current evidence was inconclusive. Sensitivity analysis by removing one trial (Klarin et al., 2008) showed similar results to the overall analysis (RR = 0.96, 95% CI = 0.73–1.26; *P* = 0.78).

### Secondary outcome 1e: hospital mortality

**Figure 11:**
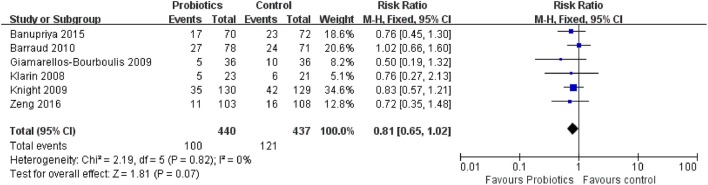
Forest plot of incidence of hospital mortality.

**Figure 12:**
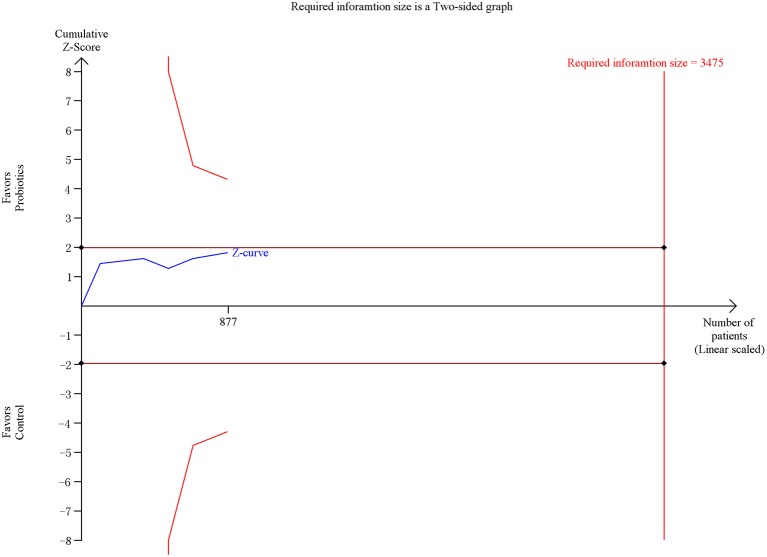
Trial sequential analysis of hospital mortality.

Six trials contacting 877 patients reported the ICU mortality data. The meta-analysis of these six trials indicated no significant difference in hospital mortality in patients exposed to probiotics based on fixed-effects model (RR = 0.81, 95% CI = 0.65–1.02; *P* = 0.07), as shown in Figure 11. No evidence of between-study heterogeneity was detected (*P* = 0.82, *I*^2^ = 0%). The TSA adjusted 95% CI was ranged from 0.49 to 1.33. The TSA result showed that 877 (25.2%) of the RIS of 3,475 patients was accrued. The cumulative z-curve crossed neither the conventional boundary for benefit nor the trial sequential futility boundary for benefit (Figure 12), revealing that the current evidence was inconclusive. Sensitivity analysis by removing two trials (Klarin et al., 2008; Banupriya et al., 2015) showed similar results to the overall analysis (RR = 0.83, 95% CI = 0.64–1.07; *P* = 0.15)

### Tertiary outcome 1a: diarrhea

**Figure 13:**
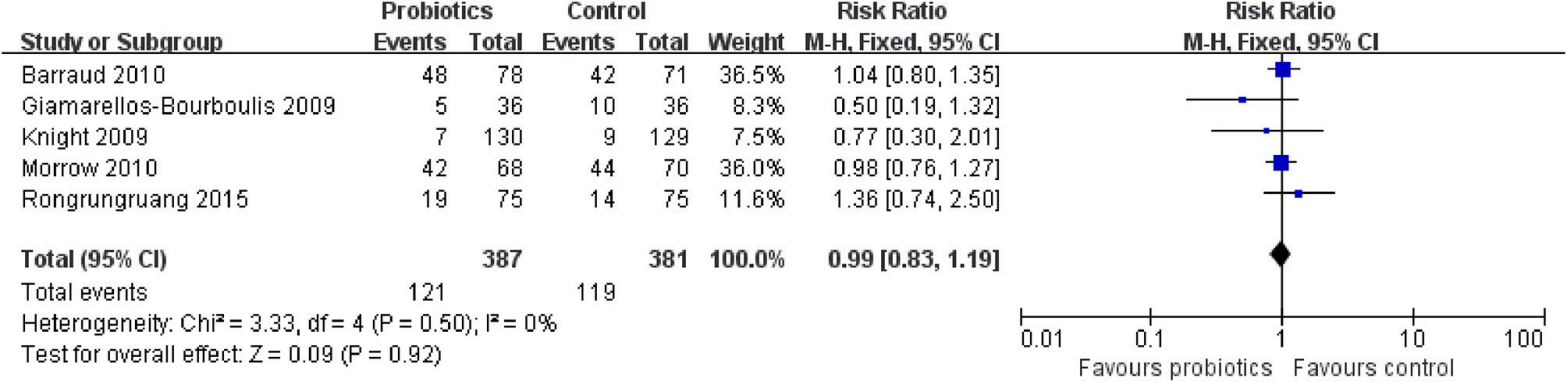
Trial sequential analysis of diarrhea.

Five trials with 768 patients reported the diarrhea data. The meta-analysis of these six trials showed no significant difference in diarrhea in patients exposed to probiotics based on fixed-effects model (RR = 0.99, 95% CI = 0.83–1.19; *P* = 0.92), as presented in Figure 13. No evidence of between-study heterogeneity was detected (*P* = 0.50, *I*^2^ = 0%).

### Tertiary outcome 1b: length of ICU stay

**Figure 14:**
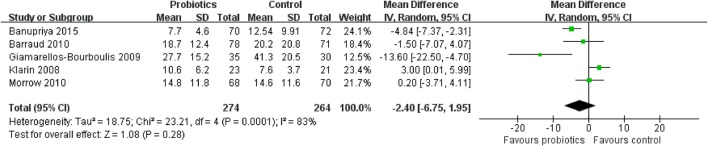
Forest plot of incidence of length of ICU stay.

Five trials including 538 patients reported the length of ICU stay. The meta-analysis of these six trials showed no significant difference in length of ICU stay in patients exposed to probiotics based on random-effects model (MD = −2.40 days, 95% CI = −6.75 to 1.95; *P* = 0.28), as shown in Figure 14. Moderate to high between-study heterogeneity was detected (*P* = 0.0001, *I*^2^ = 83%). Sensitivity analysis by removing two trials (Klarin et al., 2008; Banupriya et al., 2015) showed similar results to the overall analysis (MD = −3.88 days, 95% CI = −10.51 to 2.76; *P* = 0.25).

### Tertiary outcome 1c: length of hospital stay

**Figure 15:**
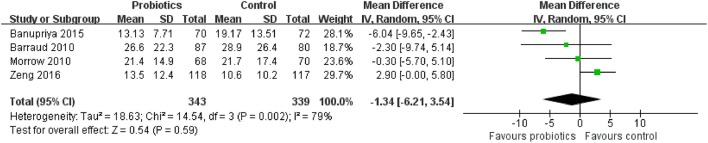
Forest plot of incidence of length of hospital stay.

Four trials with 682 patients reported the length of hospital stay. The meta-analysis of these six trials showed no significant difference in length of hospital stay in patients exposed to probiotics based on random-effects model (MD = −1.34 days, 95% CI = −6.21 to 3.54; *P* = 0.59), as displayed in Figure 15. Moderate to high between-study heterogeneity was detected (*P* = 0.002, *I*^2^ = 79%). Sensitivity analysis by removing one trial (Banupriya et al., 2015) showed similar results to the overall analysis (MD = 1.47 days, 95% CI = −1.30 to 4.25; *P* = 0.30).

### Tertiary outcome 1d: duration of mechanical ventilation

Four trials involving 512 patients reported the duration of mechanical ventilation. The meta-analysis of these six trials showed no significant difference in duration of mechanical ventilation in patients exposed to probiotics based on random-effects model (MD = −3.32 days, 95% CI = −6.74 to 0.09; *P* = 0.06), as presented in Supplementary Figure 1. Moderate to high between-study heterogeneity was detected (*P* = 0.0006, *I*^2^ = 83%). Sensitivity analysis by removing one trial (Banupriya et al., 2015) showed similar results to the overall analysis (MD = −3.32 days, 95% CI = −8.03 to 1.38; *P* = 0.17).

## Discussion

To date, the present meta-analysis is the largest and most updated evaluation of the overall effects of probiotics on preventing VAP in mechanically ventilated patients. Based on the analysis of 13 RCTs involving 1,969 patients, we found that probiotics were significantly associated with a decreased risk of VAP in mechanically ventilated patients, which was confirmed by TSA that the result of TSA showed that the cumulative Z-curve of incidence of VAP surpassed the trial sequential monitory boundary. Compared to the standard statistical analysis of meta-analysis, the results of TSA can adjust the false positives or false negatives. No significant association was observed in terms of 90-day mortality, overall mortality, 28-day mortality, ICU mortality, hospital mortality, diarrhea, length of ICU stay, length of hospital stay, and duration of mechanical ventilation.

VAP is currently the second most common nosocomial infection in America and the most prevalent ICU-acquired infection worldwide. In addition, it is a costly healthcare-associated infection. Rello et al. (2002) suggested that VAP might lead to an additional 40,000 dollar in hospital charges per patient. Branch-Elliman et al. (2015) developed a cost-benefit model to determine the most cost-effective strategy for prevention of VAP and examined a total of 120 unique combinations of VAP prevention strategies. They documented that the application of prophylactic probiotics and subglottic endotracheal tubes was cost-effective for prevention of VAP from the perspective of societal and hospital (Branch-Elliman et al., 2015). Combined the results of our present meta-analysis, we concluded that implementation of probiotics for prevention of VAP in mechanically ventilated patients had the potential to improve the incidence of VAP.

On the topic of VAP prevention in mechanically ventilated patients, four meta-analyses had been performed to evaluate the effectiveness of probiotics (Siempos et al., 2010; Gu et al., 2012; Wang et al., 2013; Bo et al., 2014). Siempos et al. (2010) and Wang et al. (2013) identified five trials, but they yielded an opposite conclusion. Besides, Gu et al. (2012) obtained seven trials and Bo et al. (2014) included eight trials. Compared with the previous meta-analyses, our meta-analysis was largest and most updated, involving 13 trials and 1,969 patients. The results of present meta-analysis were consistent with the two previous meta-analyses (Siempos et al., 2010; Bo et al., 2014), which suggested that probiotics were associated with decreased risk of VAP in mechanically ventilated patients. Furthermore, the present meta-analysis performed a further analysis to confirm the conclusion. According to the results of TSA, Z-curve of the incidence of VAP surpassed the trial sequential monitoring boundary, indicating that the result of incidence of VAP was true positive. The effect of probiotics in critically ill patients has been evaluated in several studies (Jacobi et al., 2011; Liu et al., 2012; Petrof et al., 2012; Barraud et al., 2013; Manzanares et al., 2016). They all supported that the use of probiotics could reduce the risk of infection for critically ill patients, including VAP. Therefore, the application of probiotics for VAP prevention should be recommended in clinical practice in the current healthcare circumstance.

Several limitations should be taken into consideration when interpreting the results from the present meta-analysis. First, the quality of the included trials relatively low. As shown in Figure 2, even though most of trials adequately reported the methodology, several domains still got “unclear” due to insufficient information in their studies. Second, owing to limited number of included trials, we failed to detect the publication bias, which inevitably affected the precision of our findings. Furthermore, even though we comprehensively searched the databases, the gray literature was not collected. Third, the significant between-study heterogeneity was detected, which might influence the validity of the meta-analysis. The heterogeneity might be derived from the species and dosage of probiotics as well as timing of administration. Ultimately, even though the present meta-analysis is the largest study on this topic, the sample size of the meta-analysis was not large enough. For primary outcome (incidence of VAP), 62.9% of the RIS was accrued and but the cumulative Z-curve has surpassed the trial sequential monitory boundary. For secondary outcomes, however, the cumulative Z-curves neither crossed the futility boundary nor reached RIS. Only 32.0, 15.5, and 25.2% of the RISs were accrued for overall mortality, ICU mortality, and hospital mortality, respectively. Therefore, further trials are needed to verify the conclusion.

In this meta-analysis, we found that probiotics could reduce the incidence of VAP in mechanically ventilated patients. It seems likely that probiotics provide clinical benefits for mechanically ventilated patients. Large sample size and high quality RCTs are needed to further evaluate the effect of probiotics on preventing VAP in mechanically ventilated patients. However, the TSA results of overall mortality, ICU mortality, and hospital mortality showed that there might be false-negative outcomes. Therefore, further trials warranted to identify the value of probiotics in mechanically ventilated patients in future.

## Author contributions

HW and XZ conceived and designed the study. HW, JL, ZM, and YF participated in study selection, data extraction. HW, CW, and XR performed statistical analysis. HW and XZ were involved in manuscript drafting and revision. All authors approved the final manuscript for submission and publication.

### Conflict of interest statement

The authors declare that the research was conducted in the absence of any commercial or financial relationships that could be construed as a potential conflict of interest.

## Supplementary material

Supplementary Figure 1 Forest plot of incidence of duration of mechanical ventilation.
