# Supplementary figures and images for: Probiotics for Preventing Ventilator-Associated Pneumonia in Mechanically Ventilated Patients: A Meta-Analysis with Trial Sequential Analysis
Source: Front Pharmacol. 2017 Oct 9;8:717. doi: 10.3389/fphar.2017.00717 (PMC5640711; doi:10.3389/fphar.2017.00717)

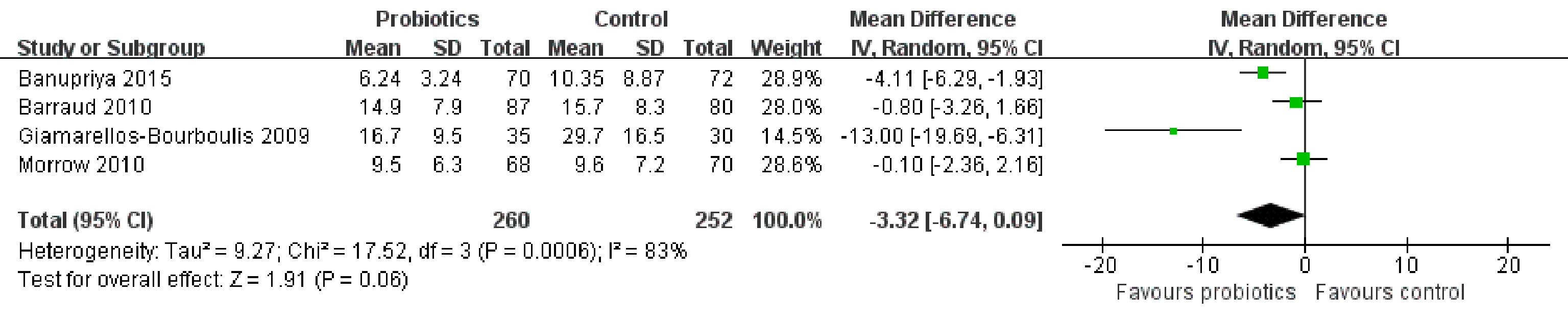

Supplement: Supplementary Figure 1 — Forest plot of incidence of duration of mechanical ventilation. [file Image1.TIF]
